# Development of Graves’ disease in a patient with lymphocytic hypophysitis following glucocorticoid treatment

**DOI:** 10.1530/EDM-24-0145

**Published:** 2025-06-25

**Authors:** Yuka Ono, Norio Wada, Shuhei Baba, Hajime Sugawara, Arina Miyoshi, Shinji Obara

**Affiliations:** ^1^ Clinical Training Center, Sapporo City General Hospital, Sapporo, Japan; ^2^ Department of Diabetes and Endocrinology, Sapporo City General Hospital, Sapporo, Japan

**Keywords:** lymphocytic hypophysitis, Graves’ disease, glucocorticoids, pregnancy

## Abstract

**Summary:**

We report the case of a 41-year-old Japanese woman with visual field disturbances during late pregnancy. At 39 weeks of gestation, she was diagnosed with bitemporal hemianopsia at the ophthalmology department. An MRI revealed a symmetrical pituitary gland enlargement, compressing the optic chiasm. An emergency cesarean section was performed immediately, resulting in the delivery of a male infant weighing 3,112 grams. Laboratory tests indicated low serum free thyroxine (T4), thyroid-stimulating hormone (TSH), cortisol, luteinizing hormone, and follicle-stimulating hormone. The patient was clinically diagnosed with lymphocytic hypophysitis (LHy). Due to her visual field impairment, she was administered 60 mg of prednisolone daily. After 2 days, her visual field impairment improved rapidly, leading to a gradual tapering of the dose. Six months after treatment initiation, an MRI showed shrinkage of the pituitary gland. Her prednisolone dose was reduced to 5 mg daily, and she was switched to hydrocortisone at 15 mg daily. Twelve months after starting treatment, the patient developed thyrotoxicosis. Testing revealed a positive TSH receptor antibody, resulting in a diagnosis of Graves’ disease (GD). Treatment with thiamazole (15 mg daily) and potassium iodide (76 mg daily) was initiated, and her thyroid function normalized after 2 months. LHy is believed to have an autoimmune mechanism and is frequently associated with other autoimmune diseases; however, the development of GD is rare. Development of Graves’ disease should be considered in patients with LHy, particularly during the postpartum period and the glucocorticoid treatment process.

**Learning points:**

- Females with lymphocytic hypophysitis often experience local symptoms, such as visual field disorders, when pregnant.
- This condition is frequently associated with autoimmune diseases, particularly autoimmune thyroid disorders. However, reports explicitly linking it to Graves’ disease have been limited.
- The postpartum period is considered a trigger of the onset of Graves’ disease. In addition, the high-dose glucocorticoid treatment and its tapering may affect it.

## Introduction

Lymphocytic hypophysitis (LHy) is the most prevalent chronic inflammatory disease that affects the pituitary gland. Initially, this inflammation was believed to be confined to the anterior pituitary gland, leading to its previous designation as lymphocytic adenohypophysitis (LAH). However, it has been recognized that the inflammation can also involve the posterior pituitary gland and the pituitary stalk, called lymphocytic infundibulo-neurohypophysitis (LINH) (1).

LAH is significantly associated with pregnancy, as approximately 50% of female patients develop the condition during pregnancy or after childbirth (1). LHy is believed to originate from autoimmune processes and often co-occurs with other autoimmune conditions such as thyroid disease (2). However, reports of cases where Graves’ disease (GD) is associated with LHy are sporadic. Here, we present a case of LHy that developed late in pregnancy and progressed to GD during glucocorticoid treatment.

## Case presentation

We report the case of a 41-year-old Japanese woman who developed visual field defects during her pregnancy. Her obstetric history included two previous pregnancies, but she had not given birth before. She began noticing visual field issues around 35 weeks of gestation. At 39 weeks of gestation, she was diagnosed with bitemporal hemianopsia at the ophthalmology department. On the same day, she visited our obstetrics department and underwent an MRI scan, which revealed an enlarged pituitary gland. An emergency cesarean section was performed the following day, leading to the delivery of a male infant weighing 3,112 grams, with an Apgar score of 8/9.

The day after delivery, she was referred to the endocrinology department. Upon admission, her height was 152 cm, weight was 63 kg, pulse rate was 67 beats per minute, and blood pressure was 109/66 mmHg. Bilateral visual field defects were noted, and a mild goiter was palpable.

## Investigation

**Figure 1:**
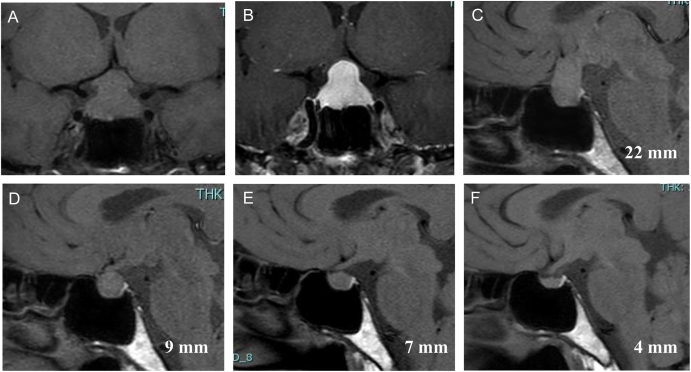
Temporal evolution of MRI findings on the pituitary gland. T1-weighted image, frontal (A) and sagittal (C) views taken 3 days postpartum. Gadolinium-enhanced image, frontal view (B), 3 days postpartum. T1-weighted image, sagittal view captured at 3 months (D), 6 months (E), and 17 months (F) after the start of treatment. The height of the pituitary gland was indicated in MRI images in (C), (D), (E), and (F).

Blood tests conducted the day after delivery indicated a normal plasma adrenocorticotropic hormone (ACTH) level of 12.8 pg/mL but a low serum cortisol level of 1.7 μg/dL. The free triiodothyronine (FT3) level was within the normal range at 2.39 pg/mL, while both the free thyroxine (FT4) and thyroid-stimulating hormone (TSH) levels were low, measuring 0.40 ng/dL and 0.02 μIU/mL, respectively. Stimulation tests using ACTH-releasing, TSH-releasing, and LH-releasing hormones revealed a responsive ACTH and cortisol response but low responses for TSH, LH, and follicle-stimulating hormone (FSH). A contrast-enhanced MRI showed symmetrical pituitary gland enlargement with dimensions of 22 × 19 × 11 mm (height x width x length), compressing the optic chiasm, with homogeneous contrast enhancement (Fig. 1A and B). These findings were suggestive of an LHy. Antithyroid globulin antibodies were negative, measuring 10.8 IU/mL, while anti-TPO antibodies were positive at 4.6 IU/mL. A thyroid ultrasound revealed mild internal echogenic heterogeneity. Human leukocyte antigen (HLA) typing identified the following alleles: A2, A33, B44, B46, Cw1, Cw14, DR8, DR13, DQ6, DQ6, DPw2, and DPw4. The specific DNA types identified were A*02:07, A*33:03, B*44:03, B*46:01, Cw*01:02, Cw*14:03, DRB1*08:03/23, DRB1*13:02, DQA1*01:02, DQA1*01:03, DQB1*06:01, DQB1*06:04, DPA1*01:03, DPA1*02:02, DPB1*02:02, and DPB1*04:01.

## Treatment

Due to visual field defects, oral prednisolone was administered at a dosage of 60 mg per day on the third day after delivery. The visual field defects improved within 2 days. Follow-up MRI scans showed a gradual reduction in pituitary enlargement (refer to Fig. 1C, D, E), reducing the prednisolone dose to 5 mg daily. Thyroid hormone replacement was not performed for the central hypothyroidism, and the thyroid functions had been followed up. Although LH and FSH levels were low during the perinatal period, menstruation occurred 10 months after delivery.

Six months after starting treatment, the 5 mg per day prednisolone dose was replaced with 15 mg per day of hydrocortisone, which was then gradually tapered down to 5 mg per day. Seventeen months after the treatment began, an MRI showed normalization of pituitary morphology (see Fig. 1F), and hydrocortisone was discontinued the following month.

## Follow-up and results

The results of the thyroid function tests are displayed in Table 1. Twelve months after starting treatment for LHy, the patient began to experience palpitations, excessive sweating, and weight loss. Her FT3 level rose to 14.30 pg/mL, FT4 to 4.33 ng/dL, and TSH was suppressed to less than 0.01 μIU/mL. Initially, TSH receptor antibodies (TRAb) were negative at 1.6 IU/L, and the patient was monitored without treatment. However, 1.5 months later, her FT3 level increased further to 18.30 pg/mL, FT4 to 5.12 ng/dL, and TSH receptor antibody became positive at 7.0 IU/L. The patient was subsequently diagnosed with GD and started on thiamazole (MMI) at a dosage of 15 mg and potassium iodide at 76 mg. A thyroid ultrasound indicated decreased internal echogenicity and increased blood flow. Two months after beginning treatment with MMI and potassium iodide, her FT3 and FT4 levels returned to normal.

**Table 1:** Progression of thyroid-related laboratory tests.

	Time after delivery							Reference range
	1 day	1 month	3 months	6 months	12 months	13.5 months	16 months	
Treatment	-							
PSL		30 mg	10 mg	5 mg				
HC					15 mg	15 mg	5 mg	
MMI						15 mg	15 mg	
KI						76 mg	38 mg	
FT3, pg/mL	2.39	1.62	1.77	2.27	14.30	18.30	2.40	2.30–4.00
FT4, ng/dL	0.40	0.93	1.08	1.02	4.33	5.12	0.46	0.90–1.70
TSH, μIU/mL	0.02	0.21	0.24	0.21	<0.01	<0.01	1.04	0.62–4.23
TRAb, IU/L	-	-	-	-	1.6	7.0	-	<2

PSL, prednisolone; HC, hydrocortisone; MMI, thiamazole; KI, potassium iodine; FT3, free triiodothyronine; FT4, free thyroxine; TSH, thyroid-stimulating hormone; TRAb, TSH receptor antibody.

## Discussion

We present the case of a patient who developed LHy during the late stages of pregnancy, which was accompanied by visual field defects. The diagnosis of LHy was made based on MRI findings and the clinical course, without the need for a pituitary biopsy. The differential diagnoses considered were pituitary neuroendocrine tumor (PitNET) and Sheehan’s syndrome. PitNET was excluded due to the diffuse, homogeneous enhancement observed on MRI and the patient’s favorable response to glucocorticoid therapy. Sheehan’s syndrome was ruled out based on the lack of peripartum hemorrhage and the onset in the prepartum period. One year after the onset of LHy, the patient developed GD as glucocorticoid therapy was tapered. The diagnosis of Graves’ disease was confirmed based on thyrotoxicosis, positive TRAb, and ultrasound findings, without a thyroid scintigraphy. In our patient, TRAb was negative in the initial test. Since TRAb has a sensitivity and specificity of approximately 99% in diagnosing Graves’ disease (3), antithyroid drugs were started after a retest 1.5 months later, when TRAb was confirmed to be positive.

LHy is believed to occur through an autoimmune mechanism and is known to frequently coexist with other autoimmune diseases, including autoimmune thyroid disorders. In a review conducted by Caturegli *et al.* of 379 cases of autoimmune hypophysitis, 68 cases (18%) were associated with other autoimmune diseases. Among these, 28 cases (7.4%) were linked to Hashimoto’s thyroiditis, and six cases (1.6%) were associated with GD (2). In a separate report by Hashimoto *et al.* 26 out of 124 cases (21.0%) of LAH were associated with autoimmune thyroiditis, while only one case (0.8%) involved GD (4).

As far as we could search, four cases of LHy (excluding drug-induced cases) associated with GD have been reported (5, 6, 7, 8). Table 2 summarizes our and prior cases.

**Table 2:** Lymphocytic hypophysitis cases associated with Graves’ disease.

	Cases				
	1	2	3	4	5
Age at onset, years	62	76	35	45	41
Sex	Male	Female	Female	Male	Female
Preceding disease	GD	LHy & GD	LHy	LHy & GD	LHy
Pregnancy-related	−	−	−	−	+
Subtype of LHy	LPH	LINH	LAH	LINH	LAH
Pituitary biopsy	+	−	+	+	−
Treatment of LHy	GC, T4, DDAVP	None	HD-GC	DDAVP	HD-GC
Treatment of GD		MMI			MMI + KI
Reference	Bayram *et al.* (5)	Yamamoto *et al.* (6)	Lidove *et al.* (7)	Yamazaki *et al.* (8)	Our case

LHy, lymphocytic hypophysitis; GD, Graves’ disease; LPH, lymphocytic panhypophysitis; LINH, lymphocytic infundibulo-neurohypophysitis; LAH, lymphocytic adenohypophysitis; GC, glucocorticoids; HD, high dose; T4, thyroxine; DDAVP, desmopressin; MMI, thiamazole; KI, potassium iodide.

Of the five reported cases, including ours, two males and three females were diagnosed with LHy associated with GD. The patients’ ages ranged from 35 to 76, with a mean age of 52. Notably, our case is the only one in which LHy developed during pregnancy or postpartum.

Regarding the subtypes of LHy, two cases, including ours, involved LAH; two cases had LINH; and one had panhypophysitis. One of the two cases with LINH presented with thickening of the pituitary stalk on MRI, although diabetes insipidus developed. Both LAH cases, including our own, were treated with high-dose glucocorticoids.

Regarding the relationship between LHy and GD, the onset of LHy and GD co-occurred in two cases. In two other cases, LHy preceded the onset of GD, while in one case, GD occurred before LHy. For both cases involving LAH, GD developed after the reduction or cessation of high-dose glucocorticoids and following delivery.

Genetic factors may contribute to the development of both LHy and GD. Many patients with LHy have HLA-DQ8 and HLA-DR53 (9). In contrast, Japanese patients with GD have a high prevalence of HLA-DRB1*04:05 and HLA-DRB1*14:03. Our patient did not demonstrate any of these HLA types (10).

Childbirth is known to influence the onset of GD potentially. Reports suggest that at least 40% of women aged 20–39 years with GD develop the condition after giving birth. In our case, GD was diagnosed approximately 1 year postpartum, indicating that childbirth may have triggered the onset of the disease (11).

In our patient and the other patient with LAH (7), GD developed after administration of high-dose glucocorticoids and tapering. However, it is unclear how the administration and tapering of high-dose glucocorticoids influence the onset of GD, because there is no evidence that indicates the high-dose glucocorticoids and these tapering affect the onset of Graves’ disease.

In conclusion, it is essential to consider the potential for the onset of GD in patients with LHy, particularly during the postpartum period and throughout the glucocorticoid treatment process.

## Declaration of interest

The authors declare that there is no conflict of interest that could be perceived as prejudicing the impartiality of the research reported.

## Funding

This study did not receive any specific grant from any funding agency in the public, commercial, or not-for-profit sector.

## Patient consent

Written informed consent for publication of their clinical details and clinical images was obtained from the patient.

## Author contribution statement

YO and NW participated in the treatment of the patient. All authors collected data, interpreted the data, and wrote the manuscript. All authors read and approved the final manuscript.
